# Effect of alternative and complementary medicine on male infertility: An umbrella review

**DOI:** 10.1002/hsr2.2118

**Published:** 2024-06-24

**Authors:** Maryam Fasanghari, Afsaneh Keramat, Mojgan Tansaz, Ashraf Moini, Reza Chaman

**Affiliations:** ^1^ Student Research Committee, School of Nursing and Midwifery Shahroud University of Medical Sciences Shahroud Iran; ^2^ Center for Health Related Social and Behavioral Sciences Research Shahroud University of Medical Sciences Shahroud Iran; ^3^ Department of Traditional Medicine, School of Traditional Medicine Shahid Beheshti University of Medical Sciences Tehran Iran; ^4^ Department of Gynecology & Obstetrics, Arash Women's Hospital Tehran University of Medical Sciences Teran Iran; ^5^ Breast Disease Research Center (BDRC) Tehran University of Medical Sciences Teran Iran; ^6^ Department of Endocrinology & Female Infertility at Reproduction Biomedicine Research Center, Royan Institute for Reproduction ACER Tehran Iran; ^7^ Department of Community Medicine, Faculty of Medicine Shahroud University of Medical Sciences Shahroud Iran

**Keywords:** acupuncture, herbal medicine, male infertility, systematic review, traditional medicine

## Abstract

**Background and Aims:**

There is increasing interest worldwide in using alternative and complementary approaches for treating male infertility. This interest has spawned a multitude of published systematic reviews and meta‐analyses. The aim of this Umbrella review was to consolidate the available evidence regarding the effect of complementary and alternative medicine on male infertility to inform clinical decision‐making processes.

**Methods:**

A comprehensive search was conducted to identify systematic reviews and meta‐analyses pertaining to the effects of complementary and alternative medicine on male infertility. This search encompassed various databases including MEDLINE, CINAHL, PubMed, Scopus, Proquest, Google Scholar, SID, EMBASE, Magiran, Cochrane Library, Iranmedex, ScienceDirect, SAGE. Subsequently, two researchers independently extracted the data from the selected meta‐analyses and systematic reviews, and evaluated their methodological quality using the assessment of multiple systematic reviews 2 (AMSTAR2).

**Results:**

This analysis encompassed 11 studies, with four originating from Iran, two from Korea and five from China. The results regarding the effectiveness of complementary and alternative medicine are controversial, indicating a need for further research. The methodological quality of the systematic reviews and meta‐analyses appraised by AMSTAR 2 was rated as low or critically low. This assessment is attributed to inadequate examination of publication biases in the reviews and a lack of discussion regarding the effect of risk of bias.

**Conclusion:**

The existing evidence regarding the effectiveness of alternative and complementary medicine in addressing male infertility is limited. Furthermore, the overall methodological quality of the published systematic reviews and meta‐analyses may have been underestimated as the use of AMSTAR2 appears to be a more precise appraisal instrument compared to its predecessor.

## 1 INTRODUCTION

Male infertility is a global health concern, necessitating effective management due to its profound effect not only on the affected couple but also on childbearing.^1^

Male infertility is typically defined as the inability of a man to impregnate a fertile female partner after 1 year of unprotected sexual intercourse.^2^ With approximately 50% of infertility cases attributed to male factors, the prominence of this issue in the field of medicine is escalating.^3^, ^4^ Evidence suggests a rising prevalence of male infertility in certain populations.^5^ The etiology of male infertility is multifaceted, encompassing abnormalities in spermatogenesis, genital system disorders or obstructions, ejaculatory dysfunction, impaired sperm motility, hormonal imbalances, and compromised immune function.^2^

Male infertility has attracted increased attention in recent years due to evidence indicating a reduction in semen quality.^2^ Semen quality plays a crucial role in male infertility as an important factor in female gamete fertility.^6^ More than 90% of male infertility cases stem from low sperm count, poor sperm quality, or a combination of both.^7^ The remaining cases can be linked to various factors such as impaired ejaculation, immune factors, hormonal imbalances, and genetic abnormalities.^8^ Environmental conditions and lifestyle factors such as tobacco and alcohol consumption, changes in sexual practices and dietary habits also affect fertility and the semen quality, leading to a diverse range of etiologies and patterns of infertility across different regions.^9^, ^10^, ^11^, ^12^, ^13^ Jorgenson and colleagues found that 20% of the youth male population had sperm concentrations below the World Health Organization's recommended threshold, while 40% of young adults failed to reach the desired sperm concentration levels.^14^

Conventional treatments such as pharmacotherapy, surgical interventions, in‐vitro fertilization (IVF), and intracytoplasmic sperm injection (ICSI), have proven effective in enabling numerous men experiencing infertility problems to achieve clinical fertility.^15^

Currently, pharmacological options for male infertility include antioxidants, hormones, hexanone theobromine, l‐carnitine (LC), and other medications.^16^ However, these treatments are associated with several disadvantages including unpredictable side effects, high costs, variable efficacy based on individual cases and poor outcomes.^17^ Other treatments are sometimes ineffective, invasive, costly or associated with adverse effects and high risks.^18^ Consequently, couples may opt for nonconventional treatment options beyond the standard modalities.^19^, ^20^, ^21^ Complementary and alternative medicine (CAM) represents one such avenue. CAM includes a diverse array of medical practices, products and systems that fall outside the realm of conventional treatments, encompassing diagnostic, therapeutic, and preventive methods. It is important to note that complementary medicine is typically employed in conjunction with standard treatments.^22^ There is an increasing interest, both in the United States and globally, in modifying lifestyle factors and integrating holistic, complementary and alternative approaches into the management of male infertility.^23^ The utilization of complementary medicine for infertility treatment has been documented in various countries, with reported usage rates in Australia (over 70%),^24^ Canada (9%–23%),^25^, ^26^ United Kingdom (40%),^27^ the United States (29%),^28^ South Korea (63.5%),^29^ Taiwan(96.1%),^30^ Lebanon (41%),^31^ Iran(around 50%)^32^ indicating a trend among patients toward exploring complementary medicine for treating infertility.^33^, ^34^

Chinese herbal medicine (CHM) has a longstanding history in China for enhancing male infertility.^2^

Studies show that infertile individuals commonly turn to CAM modalities such as acupuncture.^35^, ^36^, ^37^, ^38^ herbal medicine,^39^ yoga,^40^, ^41^ artificial sleep,^42^ homeopathy,^43^ meditation, and physical therapy.^44^

Farhood and colleagues demonstrated the efficacy of herbal remedies in treating male infertility.^45^ Similarly, Bioos and colleagues highlighted the effectiveness of a specific herb in Iranian traditional medicine for treating idiopathic male infertility.^46^ Additionally, De Souza and colleagues showed that homeopathic interventions can significantly improve sperm motility and fertility.^43^

However, both healthcare professionals and individuals resorting to complementary treatments have raised concerns regarding the lack of empirical evidence supporting the efficacy of such interventions in addressing infertility.^47^ Furthermore, studies exploring the impact of complementary treatments on infertility have yielded a wide range of outcomes.^48^ Consequently, the aim of this study was to conduct an umbrella review of systematic reviews, examining the role of complementary and alternative medicine in the context of male infertility.

## 2 METHODS

### 2.1 Protocol and registration

The protocol of this study was registered in PROSPERO with the registration number CRD42023449357 (https://www.crd.york. ac.uk/PROSPERO/).

### 2.2 Research question

The research question was to determine the current status, effectiveness, and methodological quality of systematic reviews and meta‐analyses addressing complementary and alternative medicine for male infertility.

PICO framework was structured as follows^1^: Participants: males^2^; Interventions: a range of complementary and alternative medicine modalities including acupuncture, various herbal treatments and medications, yoga, homeopathy, Ayurveda, traditional medicine, Persian medicine, Chinese traditional medicine, massage therapy, and chiropractic^3^; main outcome: sperm parameter quality^4^; study design: systematic reviews with or without meta‐analyses in clinical trials.

The main outcome index focused on sperm parameters, specifically sperm motility and concentration.

Inclusion criteria encompassed papers published in English and Persian that utilized at least one complementary and alternative medicine intervention.

The exclusion criteria comprised papers, abstracts from conference proceedings, as well as animal studies and protocols.

### 2.3 Search strategy

The search encompassed all available papers published from January 2014 to March 2023 utilizing a specific set of keywords.

This study conducted searches across various databases, imposing language restrictions for English and Persian, including: MEDLINE, CINAHL, PubMed, Scopus, ProQuest, Google Scholar, SID, EMBASE, Magiran, Cochrane Library, Iranmedex, ScienceDirect, and SAGE.

Search strategies for each database were tailored using a diverse array of keywords such as “male infertility,” “acupuncture,” “herbs,” “herbal therapy,” “medicinal plants,” “Ayurveda,” “yoga,” “homeopathy,” “traditional medicine,” “Chinese traditional medicine,” “massage therapy,” “chiropractic,” “Persian medicine,” “semen quality,” “systematic review,” and “semen parameters,” employing general symbols and Boolean operators to combine terms. The search strategies for each database were adapted using a diverse array of keywords such as “male infertility,” “acupuncture,” “herbs, herbal therapy, medicinal plants, Ayurveda, yoga, homeopathy, traditional medicine, Chinese traditional medicine, massage therapy, chiropractic's, Persian medicine, semen quality, systematic review, and semen parameters, using general symbols and Boolean operators for combining terms. Additional manual searches were conducted to ensure comprehensive evidence retrieval.

### 2.4 Data selection and extraction

Upon completion of the search, two researchers independently reviewed the titles and abstracts of the identified papers to select eligible studies. The full text of the selected papers was then assessed against the predetermined inclusion and exclusion criteria. In cases of disagreement, a third researcher provided input for resolution. Data extraction was independently conducted by two researchers, capturing details such as the first author's name, publication year, country of origin, number of randomized controlled trials (RCTs) included in each study, total population size, participants' characteristics, interventions, comparator groups, and outcome measures.

### 2.5 Methodological quality assessment

Two researchers independently assessed the quality of the studies using the Assessment of Multiple Systematic Reviews 2″ (AMSTAR 2), a validated 16‐item tool for appraising the methodological quality of systematic reviews.^49^ Each item in the AMSTAR 2 tool was evaluated as “yes,” “relative yes,” and “no.” The general confidence in the study results was determined by considering the number of critical or noncritical issues, with rankings ranging from high to critically low confidence levels.^49^, ^50^

## 3 RESULTS

### 3.1 Search results

**Figure 1:**
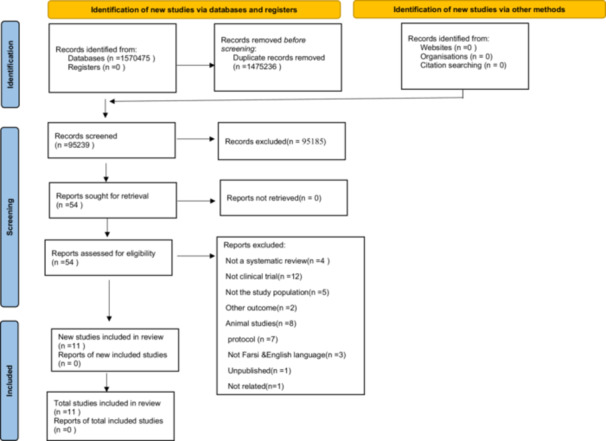
PRISMA flow diagram.

Figure 1 presents the study selection process for inclusion in the umbrella review overview. Out of 1,570,475 studies initially identified, 1,475,236 duplicates were removed. Following the screening of titles and abstracts from the remaining 95,239 studies, 95,185 studies were excluded. Subsequently, out of the 54 studies that underwent full‐text review, 43 were excluded for different reasons including noncompliance with the study design criteria. Ultimately, 11 studies were included.^51^, ^52^, ^53^, ^54^, ^55^, ^56^, ^57^, ^58^, ^59^, ^60^, ^61^ Figure 1 illustrates the PRISMA flowchart depicting the selection of published systematic review studies.

Out of the 11 studies identified, one was conducted in 2014, two in 2018, one in 2019, one in 2020, two in 2021, three in 2022, and one has been published up to March 2023. The majority of studies carried out in 2022, indicating a rising trend. The proportion of published articles is as follows: 18% in Korea, 45.5% in China, and 36.5% in Iran.

### 3.2 Characteristics of included studies

Table 1 presents the main features of the included studies. Seven systematic reviews conducted meta‐analyses,^51^, ^53^, ^54^, ^55^, ^59^, ^60^, ^61^ while four did not performed meta‐analyses.^52^, ^56^, ^57^, ^58^ The publication dates of systematic reviews ranged from 2014 to 2023. Among the studies, four studies were conducted by researchers based in Iran,^55^, ^56^, ^57^, ^58^ two in Korea,^51^, ^59^ and five in China.^52^, ^53^, ^54^, ^60^, ^61^ Additionally, 11 of the review studies were published in English. Four systematic reviews analyzed the effects of acupuncture on infertile men,^51^, ^52^, ^53^, ^54^ five assessed the effects of medicinal plants on both fertile and infertile men,^55^, ^56^, ^57^, ^58^, ^59^ and two analyzed the effects of Chinese traditional medicine on infertile men.^60^, ^61^

**Table 1:** Characteristics of the systematic reviews of complementary and alternative medicine for men.

**References/country**	**Name of the article**	**Number of RCT**	**Population/total**	**Outcome**	**Intervention**	**Comparator**	**Meta‐analysis results**
Acupuncture							
Jerng et al. (Korea)^51^	The effectiveness and safety of acupuncture for poor semen quality in infertile males: A systematic review and meta‐analysis	RCTs *n *= 4	Infertile men (*n *= 500)	Sperm motility, sperm concentration, pregnancy rate, adverse events	Acupuncture, with or without additional treatment	Placebo/sham acupuncture, no treatment, conventional therapy	Mean difference (95% CI): Sperm motility: 7.51(3,12.01) sperm concentration: 6.42(4.91, 7.92)
You et al. (China)^52^	Efficacy and safety of acupuncture for the treatment of oligoasthenozoospermia: A systematic review	RCTs *n* = 12	Patients with oligoasthenozoospermia (*n* = 1088)	Sperm concentration, sperm motility, adverse effects	Acupuncture (electroacupuncture or manual acupuncture)	Blank control, placebo, conventional drug therapy or Chinese herbal medicine	No meta‐analysis
Jia et al. (China)^53^	Acupuncture for oligospermia and asthenozoospermia: 'A systematic review and meta‐analysis	RCTs (*n* = 7)	Patients with oligospermia and asthenozoospermia (*n* = 527)	Sperm motility, sperm concentration, semen volume, the percentage of normal‐form sperm	Any types of acupuncture (manual acupuncture, scalp acupuncture, auricular acupuncture, electro‐puncture, three‐edged needle acupuncture, warm acupuncture and dry needling)	Placebo acupuncture, no treatmentcontrols, or other conventional drugs	Standard mean difference (95% CI): Sperm motility: 1.13(−0.64, 2.89) sperm concentration: 0.32(−0.27, 0.92)
Wang et al. (China)^54^	Efficacy and safety of nonpharmacologicalstrategies for the treatmentof oligoasthenospermia: A systematic review and Bayesian network meta‐analysis	RCTs (*n* = 38)	Patients with oligoasthenozoospermia (*n* = 3080)	Total effective rate, sperm concentration, sperm motility, *Sperm viability*, reproductive hormones FSH, LH, T, adverse reaction	Nondrug therapy alone (electroacupuncture, 2 Hz TEAS, 100 Hz TEAS, warming acupuncture, moxibustion, manual acupuncture, varicocelectomy, and hyperbaric oxygen)	Conventional medicine, sham intervention, or no treatment	Bayesian network meta‐analysis
Medicinal plants and herbal medicines							
Maleki et al. (Iran)^55^	A systematic review and meta‐analysis of clinical trials on saffron (*Crocus sativus*) effectiveness and safety on erectile dysfunction and semen parameters	RCTs and clinical trials (*n* = 6)	Male with erectile dysfunction, infertile men, married, diabetic male (*n* = 381)	Erectile dysfunction, sperm count, spermconcentration, sperm motility and spermmorphology	Saffron	Placebo or othermedicines	The mean percentage of sperm motility:(*p *< .001) The mean sperm concentration: (*p *= 0.1)
Roozbeh et al. (Iran)^56^	A systematic review on use of medicinal plantsfor male infertility treatment	RCTs (*n* = 20)	Fertile and infertile men (*n* = 1519)	Sperm concentration, sperm motility, sperm morphology, sperm count, Semen volume, testicular function, male reproductive system disorders	Medicinal plants (irrespective of treatmentduration and route of administration)	No treatment, treatment with placebo, and treatment with other nonherbal agents	No meta‐analysis
Ahmadian et al. (Iran)^57^	Herbal medicines for idiopathic male infertility: A systematic review	RCTs and randomized crossover trials (*n* = 14)	Men with idiopathic infertility (*n* = 1218)	Sperm count, sperm concentration, spermmotility, apoptosis level. Semen volume, vitamins A, C, E in semen, phospholipid and lipid and triglyceride levels, malondialdehyde (MDA), superoxide dismutase (SOD), Catalas, Froctos and Zinc (Zn), Cuprum (CU), Ferrum (FE), Aurum (AU), and metabolites in semen, LH, FSH, Prolactin, Testosterone, CRP, MDA	Any form of a plant, simple or compound, withany method of processing and manufacturing and any form of consumption (tablets, capsules) and prepared from any part of the plant (seeds, roots)	Placebo or medication or diet at the same time	No meta‐analysis
Shahid et al. (Iran)^58^	A systematic review on the effectiveness of herbal interventions for the treatment of male infertility	RCTs (*n* = 19)	Infertile adult males (*n* = 1398)	Sperm concentration, sperm motility, sperm morphology, totalserum FSH, total serum testosterone, conception	Any herbal intervention approved according to guidelines andmentioned in the studies	Any placebo or comparator	No meta‐analysis
Lee et al. (Korea)^59^	Maca (*Lepidium meyenii* Walp.) on semen quality parameters: A systematic review and meta‐analysis	RCTs (*n* = 5)	Infertile and healthy men (*n* = 244)	Sperm motility and sperm concentration. Sperm morphology, volume, counts.	All maca preparation types, irrespective of their origins, and trials using only maca as the mode of treatment	Any type of control, placebo‐controlled	Mean difference (95% CI): Sperm concentration: 2.22(−2.94, 7.37)
Traditional Chinese medicine							
Zhao et al. (China)^60^	The therapeutic effects of a traditional Chinese medicine formula Wuzi Yanzong Pill for the treatment of Oligoasthenozoospermia: A meta‐analysis of randomized controlled trials	RCTs (*n* = 5)	Adults with oligoasthenozoospermia (*n* = 960)	Sperm concentration, sperm motility, the normal rate of sperm morphology. the volume of semen, sperm DNA fragmentation index, the activity of acrosomal enzyme.	WZYZ pill (regardless of classical or modifed)	Placebo or vitamin control,	Mean difference (95% CI): Sperm motility: 4.57 (0.47, 8.68) Sperm concentration: 5.99 (2.12, 9.85)
Wang et al. (China)^61^	Vitamins combined with traditional Chinese medicine for male infertility: A systematic review and meta‐analysis	RCTs (*n* = 14)	Infertile men (*n* = 1488)	Pregnancy rate, sperm parameters (concentration, motility, viability)	Patients were treated with TCM	Patients in the control group were only treated with vitamins	Weighted mean difference (95% CI): Sperm motility: 13.74 (8.89, 18.60) sperm concentration: 12.91 (6.37, 19.44)

### 3.3 Methodological quality of systematic reviews

The findings of the AMSTAR 2 appraisal for each systematic review are detailed in Table 2. The results of the AMSTAR 2 analysis showed that in seven systematic reviews assessed, one exhibited a critical flaw indicative of low methodological quality. The remaining four systematic reviews displayed at least two critical flaw, categorizing them as having critically low methodological quality.

**Table 2:** The methodological quality of included SRs.

References	1	2#	3	4 #	5	6	7#	8	9#	10	11#	12	13#	14	15#	16	Quality
Jerng et al.^51^	Y	N	Y	Y	Y	Y	Y	Y	Y	N	Y	Y	Y	Y	Y	Y	Low
Maleki et al.^55^	Y	Y	Y	PY	Y	Y	N	Y	Y	N	Y	N	N	Y	N	Y	Critically low
Zhao et al.^60^	PY	Y	Y	Y	Y	Y	Y	Y	Y	N	Y	Y	Y	Y	N	Y	Low
You et al.^52^	Y	Y	Y	PY	Y	Y	Y	Y	Y	Y	Y	Y	Y	Y	N	Y	Low
Wang et al.^61^	Y	Y	Y	PY	Y	Y	Y	Y	Y	N	Y	Y	N	Y	N	Y	Critically low
Jia et al.^53^	Y	N	Y	PY	Y	Y	Y	Y	Y	Y	Y	Y	Y	Y	Y	Y	Low
Roozbeh et al.^56^	PY	Y	Y	N	Y	Y	Y	PY	Y	Y	Y	Y	Y	Y	Y	Y	Low
Lee et al.^59^	Y	Y	Y	PY	Y	Y	Y	Y	Y	Y	Y	Y	N	Y	PY	Y	Low
Ahmadian et al.^57^	Y	Y	Y	N	Y	PY	N	Y	Y	Y	Y	Y	Y	Y	Y	Y	Critically low
Shahid et al.^58^	Y	Y	Y	N	Y	Y	Y	PY	Y	N	N	N	Y	N	PY	Y	Critically low
Wang et al.^54^	Y	Y	PY	PY	Y	Y	Y	Y	Y	Y	Y	Y	N	N	Y	Y	Low
%	75	75	91	25	100	91	83	83	100	50	91	83	58	83	50	100	

None of the reviews evaluated using the AMSTAR 2 appraisal attained a rating of “high” quality, primarily because the critical criteria outlined in the AMSTAR 2 tool were not sufficiently met. Specifically, key components such as item 13 (regarding the explanation of risk of bias in meta‐analyses) and item 15 (concerning the exploration of publication biases and discussion of their effects) were inadequately addressed, resulting in the lack of any review being ranked as “high” quality (Table 2).

Among the seven critical items assessed, the systematic reviews demonstrated varying levels of performance: 75% exhibited good performance in item 2, 25% in item 4, 83% in item 7, 100% in item 9, 91% in item 11, 58% in item 13, and 50% in item 15. Concerning the nine noncritical items, all systematic reviews displayed good performance in items 5 and 16, 83% in items 8, 12, and 14, 75% in item 1, 91% in items 3 and 6, and 50% in item 10 (Table 2).

### 3.4 Acupuncture on male infertility

Four systematic reviews examined the efficacy of acupuncture in comparison to fabricated acupuncture, placebo, no treatment, as well as conventional pharmacotherapy or Chinese traditional medicine for male infertility.^51^, ^52^, ^53^, ^54^ While all studies assessed semen analysis parameters as the outcome, the effect of acupuncture on male infertility remains controversial. In a systematic review conducted by Jerng and colleagues, which compared acupuncture (manual/electric stimulation) to fabricated acupuncture, conventional treatment or no treatment in infertile men with oligozoospermia or asthenozoospermia, it was suggested that acupuncture may considerably improve sperm motility and concentration. However, the existing evidence regarding the ability of acupuncture to improve sperm concentration and motility in infertile men is deemed insufficient.^51^

In 2019, a systematic review conducted by You and colleagues compared the efficacy of acupuncture (electric or manual) with control alone, placebo, conventional pharmacotherapy, or Chinese herbs for the treatment of oligostenospermia. The review suggested that acupuncture can have superior therapeutic effects over other treatment methods for this condition. However, the clinical evidence supporting the effects of acupuncture on oligostenospermia is not conclusive, and more robust RCTs are needed for confirmation.^52^

In another systematic review by Jia and colleagues, the comparison of acupuncture alone with fabricated acupuncture or conventional pharmacotherapy did not show clear evidence of superiority in improving sperm motility (SMD = 1.13, 95% CI: 0.64–2.89) and sperm concentration (SMD = 0.32, 95% CI: 0.27–0.92). The current evidence is insufficient for drawing definitive conclusions.^53^

Furthermore, in a systematic review by Wang and colleagues, that evaluated nonpharmacological interventions against conventional therapy, fabricated intervention, or no treatment, it was found that nonpharmacological interventions were more effective than fabricated interventions or lack of treatment in improving overall efficacy and sperm concentration. Warm acupuncture was highlighted as potentially the most effective treatment for enhancing total effect and sperm concentration, while electric acupuncture could lead to the best improvement in sperm motility. The review concluded that nonpharmacological treatments for oligoasthenospermia demonstrate favorable clinical efficacy. As such, these treatment approaches can be recommended based on individual circumstances.

However, due to limitations in the quality of the included studies, further research is needed to validate these findings.^54^

### 3.5 Herbs on male infertility

Five systematic reviews reported effects of herbal drugs compared to placebo in both fertile and infertile men.^55^, ^56^, ^57^, ^58^, ^59^ The effects of herbal drugs on male infertility remains controversial.

In a systematic review conducted by Maleki and colleagues, the efficacy of oral saffron consumption on semen parameters and impaired ejaculation function was evaluated. This review included six RCTs, with two of them specifically focusing on the effects of saffron on semen parameters in infertile men. The results from these two RCTs regarding semen parameters were contradictory. In one RCT, saffron demonstrated a significant improvement in the morphology and motility of sperm from class A to class C, but did not show a significant impact on sperm count. Conversely, the second RCT found that saffron did not lead to any significant changes in mean sperm count, sperm concentration, sperm motility, or sperm morphology.^55^

In a study by Roozbeh and colleagues, which compared herbal drugs with no treatment, placebo, and nonherbal treatments, it was observed that herbal drugs resulted in improvement in semen volume and sperm quality in infertile men. For developing a novel approach in the management of male infertility, further clinical experiments are essential to establish the optimal dosage and duration of herbal drug treatment, as well as to assess any potential side effects associated with such interventions.^56^

The results from the systematic review conducted by Ahmadian and colleagues which compared the plant‐based interventions against placebo, drugs or dietary interventions, showed that certain plants can be effective in treating male idiopathic infertility. However, more precise clinical studies with adequate sample sizes and extended durations are necessary to yield more robust and reliable results.^57^

In the systematic review by Shahid and colleagues, which assessed various plant‐based interventions following established guidelines against placebos or eligible comparator, it was suggested that herbs especially *Withania somnifera* and Hochu‐ekki‐ could potentially be effective in managing male infertility. However, there is a need for future experimental studies to formulate improved therapeutic strategies for enhancing treatment outcomes.^58^

In a study by Lee and colleagues investigating the effects of Maca in comparison with various controls or placebos, it was noted that the effect of Maca on qualitative semen parameters in both infertile and healthy men remains uncertain. The limited number of RCTs and small sample sizes hinder the ability to draw a definitive conclusions regarding the efficacy of Maca in this context.^59^

### 3.6 Chinese traditional medicine on male infertility

Two systematic reviews reported the findings of Chinese traditional medicine interventions compared to placebos and vitamins in infertile men.^60^, ^61^ The effect of Chinese traditional medicine on male infertility remains controversial. The results of systematic review conducted by Zhao and colleagues, which compared the traditional Chinese medicine Formula Wuzi Yanzong Pill against placebos or vitamins, indicated that WZYZ pill can increase sperm concentration, improve sperm motility, enhance the activity of acrosome enzyme, and reduce sperm DNA fragmentation in individuals with. oligoasthenozoospermia However, the inherent limitations of the studies included in the review pose challenges in drawing a definite conclusion.^60^

The systematic review conducted by Wang and colleagues showed that in comparison with vitamin E or vitamin C+E alone, the combination of Chinese traditional medicine with vitamins would enhance sperm concentration, sperm motility, sperm viability, semen liquefaction time, acrosome enzyme activity, and overall fertility outcomes in individuals with male infertility. This combination represent a novel regimen warranting further exploration through larger, and longer‐term multicenter clinical studies to discover the best parameters.^61^

## 4 DISCUSSION

The utilization of complementary medicine in the treatment of infertility is a subject of growing interest within the research community. However, the assessment of the effects of complementary and alternative medicine on male infertility remains a contentious issue. This umbrella review aimed to investigate the effect of complementary and alternative medicine on semen analysis parameters, specifically focusing on sperm motility and concentration. This study encompassed an analysis of 11 systematic reviews published from January 2014 to March 2023, with each review comprising 4–38 RCTs involving 244–3080 participants. In most studies, acupuncture was compared with fabricated acupuncture, placebo and no treatment, conventional pharmacotherapy, or Chinese traditional medicine in infertile men. Some systematic reviews showed that acupuncture could affect the sperm motility and concentration. However, the current clinical evidence regarding the efficacy of acupuncture in this context is deemed insufficient or inconclusive, necessitating further confirmation due to study limitations.^51^, ^52^, ^54^ Contrarily, a separate systematic review indicated that acupuncture had no impact on improving the sperm motility and concentration.^53^

Numerous systematic reviews comparing the efficacy of herbal interventions against placebo, no treatment, and pharmacotherapy have reported contradictory findings. While certain reviews have indicated the potential effectiveness of specific medicinal plants in improving sperm quality. Nevertheless, future experimental studies are required to facilitate the development of more reliable pharmacotherapies.^56^, ^57^, ^58^ Conversely, some systematic reviews have reported inconclusive and conflicting results regarding the impact of herbal interventions on sperm quality.^55^, ^59^

The assessment of the effects of Chinese traditional medicine on male infertility through systematic reviews remains controversial. Despite indications from systematic reviews suggesting the potential efficacy of Chinese traditional medicine in improving sperm quality, the presence of inherent limitations in the underlying studies have prevented the ability to draw definitive conclusions, underscoring the necessity for more clinical studies.^60^, ^61^

It seems that the results of the meta‐analysis of traditional Chinese medicine are more successful than acupuncture and herbal medicine. Insufficient research exists on the effectiveness of complementary and alternative medicine approaches in treating male infertility, underscoring the necessity for further investigations to elucidate the potential positive effects of such methods on semen analysis parameters. The current study highlights the ambiguous and conflicting findings from existing research on complementary and alternative medicine in male infertility, with a majority of studies advocating for additional research to clarify the outcomes.

### 4.1 Methodological quality of the included systematic reviews

This umbrella review contributes valuable insights to inform decision‐making about the utilization of complementary medicine in the context of male infertility for healthcare practitioners and policymakers. Across the majority of systematic reviews analyzed, the methodological quality was low, with notable deficiencies identified in key areas outlined in AMSTAR2, specifically pertaining to items Q13 and Q15. Item 13 of AMSTAR2 evaluates whether the discussion addresses the effect of risk of bias on the results despite conducting a meta‐analysis, only three systematic reviews incorporating this aspect in their reports. Similarly, item 15 appraises the thoroughness of examining publication biases and discussing their implications, a practice observed in only six systematic reviews. Six systematic reviews had reported it. Given that AMSTAR2 is a more precise appraisal tool compared to its predecessor, it is imperative to interpret the appraisal results with caution, recognizing that the methodological quality of the included systematic reviews and meta‐analyses may have been underestimated.

### 4.2 Limitations

This umbrella review is subject to several limitations. First, the overall quality of the included studies, as appraised precisely by the latest AMSTAR 2 was deemed to be low. Future systematic reviews and meta‐analyses should accurately evaluate the specific items of AMSTAR 2. Second, drawing a definitive conclusion regarding the effectiveness of complementary and alternative medicine on male infertility was challenging due to variability in the type of complementary or alternative medicine and the timing of intervention in RCTs across the included systematic reviews. Third, the exclusion of non‐English studies, attributable to resource constraints and difficulties associated with translation, may have impacted the current analysis. The inclusion of data from non‐English reviews may potentially change the importance of the findings related to different interventions of complementary and alternative medicine on male infertility. Thus, the effectiveness of complementary and alternative medicine in this context remains inconclusive.

## 5 CONCLUSION

From January 2014 to March 2023, a total of 11 systematic reviews and meta‐analyses were published, encompassing 4–38 clinical trials and involving 244–3080 participants in each review. The investigation of semen analysis parameters highlighted the need for further evidence for determining the impact of complementary and alternative medicine on male infertility.

Notably, when subjected to the more stringent methodological assessment of AMSTAR 2, the methodological quality of the systematic reviews and meta‐analyses was found to be suboptimal. This was attributed to the lack of explicit discussion concerning the risk of bias in the meta‐analyses, as well as inadequate exploration of publication biases. When doing systematic reviews and meta‐analyses in the future, caution is needed to prevent from underestimation of methodological quality due to precise appraisal criteria of the relatively new instrument AMSTAR2. Given the variety of interventions and different types of acupuncture and herbal drugs, unfortunately there is no sufficient evidence for the effectiveness of complementary and alternative medicine. Further studies are required to determine whether complementary and alternative medicine has positive effects on semen analysis parameters.

## AUTHOR CONTRIBUTIONS

**Maryam Fasanghari**: Supervision; conceptualization; methodology; writing—review & editing; project administration; formal analysis; validation; investigation. **Afsaneh Keramat**: Supervision; writing—review & editing; conceptualization; project administration; formal analysis; validation; investigation; methodology. **Mojgan Tansaz**: Supervision; writing—review & editing; conceptualization; project administration; validation; formal analysis. **Ashraf Moini**: Supervision; methodology; conceptualization. **Reza Chaman**: Supervision; validation; formal analysis; methodology; investigation.

## CONFLICT OF INTEREST STATEMENT

The authors declare no conflict of interest.

## TRANSPARENCY STATEMENT

The lead author Reza Chaman affirms that this manuscript is an honest, accurate, and transparent account of the study being reported; that no important aspects of the study have been omitted; and that any discrepancies from the study as planned (and, if relevant, registered) have been explained.

## Data Availability

Not applicable.
